# Epigenome-Wide Association Study of Cognitive Functioning in Middle-Aged Monozygotic Twins

**DOI:** 10.3389/fnagi.2017.00413

**Published:** 2017-12-12

**Authors:** Anna Starnawska, Qihua Tan, Matt McGue, Ole Mors, Anders D. Børglum, Kaare Christensen, Mette Nyegaard, Lene Christiansen

**Affiliations:** ^1^The Lundbeck Foundation Initiative for Integrative Psychiatric Research, iPSYCH, Aarhus, Denmark; ^2^Department of Biomedicine, Aarhus University, Aarhus, Denmark; ^3^Center for Integrative Sequencing, iSEQ, Aarhus University, Aarhus, Denmark; ^4^The Danish Twin Registry, Institute of Public Health, University of Southern Denmark, Odense, Denmark; ^5^Department of Clinical Genetics, Odense University Hospital, Odense, Denmark; ^6^Department of Psychology, University of Minnesota, Minneapolis, MN, United States; ^7^Psychosis Research Unit, Aarhus University Hospital, Risskov, Denmark; ^8^The Danish Aging Research Center, Institute of Public Health, University of Southern Denmark, Odense, Denmark; ^9^Department of Clinical Biochemistry and Pharmacology, Odense University Hospital, Odense, Denmark

**Keywords:** epigenome-wide association study, DNA methylation, epigenetic epidemiology, cognition, cognitive aging, whole blood, monozygotic twins

## Abstract

As the world's population ages, the age-related cognitive decline presents a great challenge to world's healthcare systems. One of the molecular mechanisms implicated in cognitive ageing is DNA methylation, an epigenetic modification known to be a key player in memory formation, maintenance, and synaptic plasticity. Using the twin design we performed an epigenome-wide association study (EWAS) in a population of 486 middle-aged monozygotic twins (mean age at follow-up 65.9, *SD* = 6.1) and correlated their blood DNA methylation to their level (cross-sectional analysis) and change in cognitive abilities over 10 years (longitudinal analysis). We identified several CpG sites where cross-sectional cognitive functioning was associated with DNA methylation levels. The top identified loci were located in *ZBTB46* (*p* = 5.84 × 10^−7^), and *TAF12* (*p* = 4.91 × 10^−7^). KEGG's enrichment analyses of the most associated findings identified “Neuroactive ligand-receptor interaction” as the most enriched pathway (*p* = 0.0098). Change in cognitive functioning over 10 years was associated with DNA methylation levels in *AGBL4* (*p* = 9.01 × 10^−7^) and *SORBS1* (*p* = 5.28 × 10^−6^), with the first gene playing an important role in neuronal survival and the latter gene implicated before in Alzheimer's disease and ischemic stroke. Our findings point to an association between changes in DNA methylation of genes related to neuronal survival and change of cognitive functioning in aging individuals.

## Introduction

The age-related cognitive decline is suggested to be caused by a combination of subtle and selective, region-specific changes resulting from alterations in dendritic morphology, cellular connectivity, Ca^2+^ homeostasis, gene expression and other factors altogether affecting neuronal plasticity (Burke and Barnes, 2006; Tripathi, 2012; Morrison and Baxter, 2014). According to monozygotic and dizygotic twin studies cognitive levels and cognitive decline are also influenced by individual's genetic background, with heritability estimates for these traits varying depending on the cognitive test applied (Swan, 1992; McGue and Christensen, 2002, 2013). However, the correlation of cognitive abilities between twins was reported to decline with age what points to non-shared environmental and stochastic effects accumulated across lifespan to influence levels of cognitive functioning in aging individuals (McCartney et al., 1990; McGue and Christensen, 2013).

Among various age-related changes taking place in the brain and multiple factors influencing them there is a growing body of evidence for a crucial role of DNA methylation in memory formation, maintenance, synaptic plasticity, and also cognitive functioning (Miller et al., 2008, 2010; Day and Sweatt, 2010; Yu et al., 2011; Roth et al., 2015; Halder et al., 2016; Maag et al., 2017). DNA methylation is an epigenetic modification known to be a powerful regulator of gene expression across tissues and lifespan (Feinberg, 2007; Jones, 2012; Lister et al., 2013; Medvedeva et al., 2014). Along the human life age-related changes in DNA methylation seem to be driven by two mechanisms: epigenetic clock and epigenetic drift, where the first phenomena describes functional age-related epigenetic changes at specific sites of the genome common across individuals, while the latter one relates to the global decrease in stability and precision of DNA methylation with age (Jones et al., 2015). These genome-wide changes in DNA methylation levels are complex and tend to increase with age in regions known for their low methylation, e.g., promoter-associated CpG islands, but decrease in regions with high DNA methylation, e.g., intergenic non-island CpG sites, thus leading to a global loss of methylation in the genome (Illingworth and Bird, 2009; Heyn et al., 2012). Maintenance of DNA methylation in the human genome across life depends mainly on two groups of enzymes: DNA methyltransferases (DNMTs), responsible for the addition of methyl group to the 5th position of cytosine, and ten-eleven translocation (TET) family enzymes that catalyze oxidation of 5-methylcytosine to forms further removed by base excision repair and substituted back with an unmodified cytosine (Chen and Riggs, 2011; Kohli and Zhang, 2013). There is a growing body of evidence linking these DNA methylation maintenance enzymes with cognitive functioning. Dnmt1 and Dnmt3a deficiency in adult mouse central neurons system was reported to cause defects in synaptic plasticity, learning and memory (Feng et al., 2010). In the mouse hippocampus cognitive decline was associated with decreased expression of *Dnmt3a2*, while rescuing its transcription levels was shown to restore cognitive functions in both young and aged mice (Oliveira et al., 2012, 2015). Tet1-deficient mice were reported to have normal brain development, but impaired spacial learning and memory. Moreover, decrease in *Tet1* expression in neural progenitor cells was associated with aberrant promoter hypermethylation of genes associated with regulation of adult neurogenesis (Zhang et al., 2013). All these studies point to the important role of DNA methylation in cognitive functioning and age-related cognitive decline.

Identification of these DNA methylation changes in an accessible tissue could allow for an early detection of decrease in cognitive functioning before more severe cognitive impairment appears, but also point us to genes involved in cognitive ageing.

In this study we aimed at investigating if blood DNA methylation signatures associate with cross-sectional level of cognitive functioning, as well as longitudinal change in cognition over a preceding 10-year period. As levels of both cross-sectional and longitudinal cognitive functioning are known to be influenced by genetic factors one of the powerful study designs to delineate epigenetic signatures associated with a phenotype of interest is using monozygotic twins (McGue and Christensen, 2013; Davies et al., 2015). Several studies reported differences in DNA methylation levels between discordant monozygotic twins to be associated with diagnosis of common disorders, such as cancer, diabetes, autoimmune diseases, as well as mental disorders (Javierre et al., 2010; Dempster et al., 2011, 2014; Heyn et al., 2013; Stefan et al., 2014; Castellani et al., 2015). We therefore performed an epigenome-wide association study (EWAS) between cognitive abilities and DNA methylation patterns in blood samples obtained from 243 pairs of monozygotic middle-aged twins, which allowed us to correct for possible genetic and shared environmental effects.

## Materials and methods

### Study population and cognitive functioning assessment

The study was performed on 486 monozygotic twins (111 female and 132 male pairs) recruited by the Danish Twin Registry as a part of The Middle Aged Danish Twin Study (MADT). The cohort is a Danish nation-wide investigation of twins randomly selected among eligible twin pairs from birth cohorts 1931–1952 (Skytthe et al., 2013). MADT was initiated in 1998, and a follow-up study was conducted in 2008-11. Whole blood samples were collected from all individuals during the follow-up visit. Zygosity for MADT cohort was assessed as described before by Christiansen and co-authors (Christiansen et al., 2003). Informed consent was obtained for all participants. Written informed consents obtained from all participants, and the surveys, including collection of blood and use of survey information, were approved by the Regional Committees on Health Research Ethics for Southern Denmark (S-VF-19980072). The present study included all monozygotic twin pairs from the MADT cohort that participated in both surveys (at the admission and at follow-up). Mean age of investigated twins at follow-up was 65.9 (*SD* = 6.1, min = 55, max = 79).

General cognitive functioning was assessed at both occasions for all twins (at the admission and at follow-up) by computing a cognitive composite score. The score was based on six brief cognitive tests (verbal fluency, immediate word recall, delayed word recall, processing speed, attention, and working memory) as previously described (McGue and Christensen, 2002). There are two reasons for using the cognitive composite score rather than the individual cognitive component scores in our analyses. First, the cognitive component scores manifest the typical positive inter-correlation that is seen among specific cognitive measures and this is taken as evidence for the existence of a general cognitive factor that underlies all the individual abilities, which is what our cognitive composite seeks to assess (McGue and Christensen, 2001). Second, it was shown that the cognitive composite score used in this study is a substantially more reliable measure of cognitive functioning over time than the individual component scores (McGue and Christensen, 2001).

Scores obtained from each cognitive test were first standardized to a mean of 0 and SD of 1 and then the 6 scores were summed to obtain the composite score. The same standardization parameters were used with both the intake and follow-up assessments to ensure the two composite scores were commeasurable. No more than one missing component was allowed and in case of individuals where two or more component scores were missing the cognitive functioning score was coded as not available. In the case where only one component measure was missing the computed cognitive functioning score was generated by prorating the available scores (i.e., by multiplying the provisional score by 6/5), as described before for this cognitive measure (Petersen et al., 2016).

### DNA methylation analysis

Whole blood samples were collected for all individuals during the follow-up assessment. Genomic DNA was extracted from buffy-coats with the use of the semi-automated salt precipitation protocol with Autopure System (Qiagen, Valencia, CA, USA). Genomic DNA (500 ng/sample) was bisulfite converted with EZ Methylation Gold Kit (Zymo Research, Irvine, CA, USA) and analyzed using the Infinium HumanMethylation450 BeadChips (Illumina, San Diego, CA, USA) array according to manufacturer's protocol. Obtained DNA methylation data was subjected to quality control with two different pipelines, combination of MethylAid (van Iterson et al., 2014) and minfi tools (Aryee et al., 2014). Probes with low bead count (<3 beads), high detection *p*-value (>0.01), zero signal, missing in >5% of samples and cross-reactive ones as reported before (Chen et al., 2013) were removed from the dataset, leaving 453,014 probes for further analysis. Data was normalized with the use of functional normalization (Fortin et al., 2014) and obtained beta values were further logit transformed.

### Blood cell composition

Blood cell counts were available for 477 individuals, where blood leukocyte subtypes (monocytes, lymphocytes, basophils, neutrophils, eosinophiles) were counted using a Coulter LH 750 Hematology Analyzer (Beckman Coulter, Woerden, The Netherlands). For the remaining nine individuals, where blood cell composition was not available, data was imputed by partial least squares regression. A regression model was first fitted based on the samples for which measurements were available, and afterwards the model was applied for prediction of missing cell counts. The model used log(cell count+1) as response and included all beta values, that were available for all samples, as covariates. Detailed procedure for cell imputation is provided on GitHub (https://github.com/mvaniterson/wbccPredictor). Further covariates included were gender, age at follow-up visit, and sentrix position (modeled by two categorical variables indicating the position in each of the two directions on the chip: Left or Right and respectively 1–6 for the other direction). All calculations were performed in R (R Core Team, 2015).

### Statistical analyses

The analyses were performed with two approaches: paired and unpaired. In the paired approach we first calculated the intra-pair difference in DNA methylation level, cross-sectional cognition (measured at follow-up) and 10-year longitudinal change of cognition, as well as difference in blood cell counts for each twin pair. Longitudinal change of cognition was calculated by subtracting the cognitive score obtained at the beginning of the study from the cognitive score at the follow-up. The null hypothesis that there was no change in cognition over 10-year period was tested with linear mixed model specifying twin pairing as random effect.

Epigenome-wide association study (EWAS) analyses were performed in four different combinations to test cross-sectional and longitudinal cognitive functioning in both within-pair (paired) and individual (unpaired) approaches. First, in the paired analyses we used linear regression models to test for an association between intra-pair DNA methylation difference and the cross-sectional cognition difference, as well as difference in longitudinal change in cognition. The models were adjusted for sex and age at the follow-up visit of investigated twin pairs, as well as for their blood cell composition difference. As a subtype of the paired EWAS analysis for cross-sectional cognition we repeated the analysis including only the 50% most discordant twins, expecting that removing the twin pairs with almost no intra-pair difference in cognition can strengthen the signal of the most associated loci with cognitive functioning.

Secondly, we performed the unpaired analyses where all twins were treated as separate individuals but controlled for dependency of observations within twin pairs in order to avoid deflated *p*-values. Association of DNA methylation with cognition at follow-up, as well as with change of cognition over time were investigated with linear mixed models in order to correct for relatedness in the sample. The unpaired analyses were also adjusted for sex, age at the follow-up visit and cell composition of the investigated individuals. This study used two types of analyses (paired and unpaired) as they involve different statistical models that differently estimate and handle confounding factors. The paired analysis works on intra-pair differences and corrects for the effects from pair-specific confounding factors (age and sex) on intra-pair differences. This aspect of the analysis is important because the intra-pair differences can increase with increasing age of the pair. Moreover, intra-pair difference can be different between male pairs and female pairs. The unpaired analysis also corrects for age and sex, but it does that for the effects on individual level of DNA methylation. The age and sex effects on intra-pair difference are ignored in this case. Additionally, as the unpaired analysis works on the individual level data it is capable of using incomplete twin pairs what increases sample size.

In this study we report all findings with unadjusted *p* < 10^−5^ to provide a better overview of the top associated findings, while the suggested genome-wide significance threshold for EWAS analyses performed on data generated with 450K DNA methylation array is *p* < 10^−6^ (Tsai and Bell, 2015).

Pathway enrichment analysis was performed using Kyoto Encyclopedia of Genes and Genomes (KEGG) and Pathway Commons enrichment with WebGestalt (Wang et al., 2013) against the genes included in the 450K DNA methylation array. *P*-values from enrichment analyses were corrected for multiple testing with Benjamini-Hochberg correction method (Benjamini and Hochberg, 1995).

## Results

DNA methylation data for all investigated individuals (*n* = 486) passed all quality control steps performed with minfi and MethylAid tools. Cognition data was available for 485 individuals at admission (mean = −0.53, *SD* = 3.71, min = −9.41, max = 10.27) and 480 individuals at follow-up (mean = −2.17, *SD* = 3.76, min = −16.75, max = 12.53). Cognitive functioning of the investigated individuals was found to decrease over the 10-year period (*p* < 2 × 10^−16^, Supplementary Figure 1). Additional data on sample demographics split by sex of investigated monozygotic twin pairs is presented in Supplementary Table 1.

For the paired analysis regressing intra-pair difference in DNA methylation on intra-pair difference in cognition at follow-up resulted in five probes with *p* < 10^−5^ with cg05867245, mapping to the Zinc Finger And BTB Domain Containing 46 gene (*ZBTB46*), being the most associated finding with *p* = 5.84 × 10^−7^ (Table 1). The probe was also found to be associated in the 50% most discordant twins analysis, however with a higher *p* = 2.45 × 10^−5^. Apart from cg05867245, three more probes (*p* < 10^−4^) were identified for cognition at follow-up in both the analysis of all twins and the 50% most discordant twins (Supplementary Table 2). Regressing intra-pair methylation differences on longitudinal change in cognition over time resulted in 11 findings with *p* < 10^−5^, with the most associated probe cg03599618 mapping to ATP/GTP Binding Protein-Like 4 gene (*AGBL4*) with *p* = 9.01 × 10^−7^.

**Table 1:** Overview of the results (*p* < 10^−5^) from the EWAS of cognitive functioning in the paired and unpaired analyses.

		**Probe**	**Estimate**	**Standard error**	***P*-value**	**Chr**	**Position (bp)**	**Gene**	**Distance to gene (bp)**	**CGI^d^ feature**
Paired analyses	Cognition at follow-up	cg05867245	0.0295	0.0057	5.84E-07	20	62402415	*ZBTB46*	NA	Body-island
		cg13336662	0.0274	0.0061	9.76E-06	8	76147299	*CRISPLD1*	25,0591	IGR^a^-open sea
		cg19312085	0.0386	0.0080	2.62E-06	1	247553836	*NLRP3*	−25,622	IGR^a^-open sea
		cg19480750	−0.0376	0.0082	6.66E-06	3	141764977	*TFDP2*	101,707	IGR^a^-open sea
		cg25537962	0.0275	0.0061	9.64E-06	6	74405564	*CD109*	NA	5′UTR^b^-shore
	Cognition at follow-up (50% most discordant)	cg04885315	0.0392	0.0076	1.00E-06	15	57731648	*CGNL1*	NA	Body-open sea
		cg07157058	−0.0630	0.0133	6.96E-06	6	7390083	*CAGE1*	NA	TSS200^c^-island
		cg09439348	0.0308	0.0062	2.23E-06	2	47305388	*C2orf61*	−8,742	IGR^a^-open sea
		cg11892307	0.0341	0.0073	8.59E-06	6	470595	*OR2J2*	−10,798	IGR^a^-open sea
		cg17261234	−0.0517	0.0110	7.22E-06	14	101925619	*DIO3OS*	−92,941	IGR^a^-island
	Longitudinal cognition change (over 10-year period)	cg02483043	−0.0194	0.0041	3.86E-06	10	134536498	*INPP5A*	NA	Body-shore
		cg03599618	0.0195	0.0039	9.01E-07	1	49361197	*AGBL4*	NA	Body-shelf
		cg05395403	−0.0282	0.0060	5.01E-06	13	28496641	*PDX1*	NA	Body-shore
		cg13630845	−0.0247	0.0054	7.73E-06	19	16682861	*SLC35E1*	NA	1stExon-island
		cg15599875	0.0366	0.0080	7.46E-06	11	60489528	*MS4A8*	22,481	IGR^a^-open sea
		cg17261234	−0.0288	0.0061	3.75E-06	14	101925619	*DIO3OS*	−92,941	IGR^a^-island
		cg17298279	−0.0179	0.0039	6.22E-06	6	158982078	*TMEM181*	NA	Body-shore
		cg18345386	−0.0221	0.0045	2.04E-06	3	62363466	*FEZF2*	8,119	IGR^a^-shore
		cg27196467	0.0495	0.0106	4.89E-06	4	21305490	*KCNIP4*	NA	Body-open sea
		cg27281093	−0.0295	0.0062	3.07E-06	22	18632404	*USP18*	NA	TSS^c^1500-shore
		cg27630540	−0.0128	0.0028	8.25E-06	4	1347166	*KIAA1530*	NA	Body-open sea
Unpaired analyses	Cognition at follow-up	cg05651282	0.0117	0.0025	2.96E-06	6	31370700	*MICA*	NA	TSS^c^1500-shore
		cg06855567	−0.0172	0.0038	9.32E-06	4	177117009	*SPATA4*	NA	TSS^c^200-shore
		cg07898057	0.0090	0.0018	4.91E-07	1	28969987	*TAF12*	NA	TSS^c^1500-shore
		cg08832744	0.0077	0.0016	2.36E-06	17	79283095	*C17orf55*	NA	TSS^c^200-shore
		cg23962175	−0.0093	0.0020	6.03E-06	14	94088604	*KIAA1409*	NA	Body-open sea
	Longitudinal cognition change (over 10-year period)	cg25421530	−0.0100	0.0022	5.28E-06	10	97200987	*SORBS1*	NA	5′UTR^b^-open sea

a *IGR, Intergenic region*;

b *UTR, Untranslated Region*;

c *TSS, Transcription Start Site*;

d *CGI, CpG Island*.

**Figure 1:**
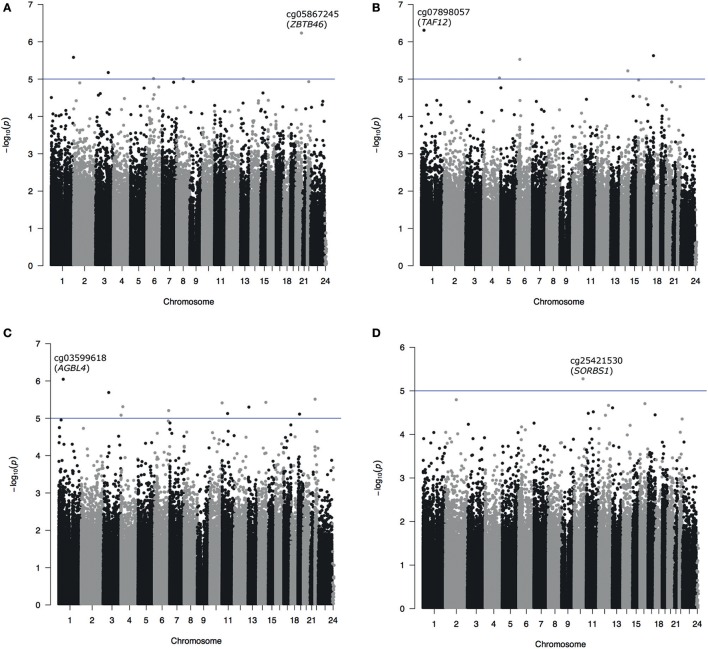
Manhattan plots from EWAS for cross-sectional (paired **A** and unpaired **B**) and longitudinal cognitive functioning (paired **C** and unpaired **D**) analyses. Probes with *p* < 10^−6^ are labeled with the probe name and annotation to the most proximal gene **(A–C)**. Due to no findings with *p* < 10^−6^ in the unpaired analysis of longitudinal cognitive functioning the most associated probe was marked on **(D)**.

In the unpaired analyses 5 CpG sites were identified with *p* < 10^−5^, with the most associated probe identified as cg07898057, annotated to TATA Box Binding Protein (TBP)-Associated Factor gene (*TAF12*) (*p* = 4.91 × 10^−7^). Unpaired analysis of change of cognition over time resulted in only one probe with *p* < 10^−5^ (cg25421530) mapping to the Sorbin And SH3 Domain Containing 1 gene (*SORBS1*) (*p* = 5.28 × 10^−6^). All probes with *p* < 10^−5^ in paired or unpaired analyses were annotated with their most proximal gene, genomic position and CpG island context and are presented together with their regression *p*-values in Table 1. Manhattan plots depicting results from all four main analyses are presented as Figures 1A–D.

In order to check if there is an overlap of results between all four EWAS analyses (with findings with *p* < 10^−4^) we prepared two Venn diagrams (Supplementary Figures 2A,B) that depict probe and gene overlap. Four probes (*p* < 10^−4^) were found to be common in the paired analyses between cross-sectional and longitudinal cognitive functioning. One probe was common in cognition at follow-up analyses between paired and unpaired analyses, and one probe was common in change of cognition analyses between paired and unpaired analyses (Table 2). Search for common genes across analyses identified the Ewing Tumor-Associated Antigen 1 (*ETAA1*), Annexin A6 (*ANXA6*), DIO3 Opposite Strand/Antisense RNA (*DIO3OS*), Transmembrane Protein 132D (*TMEM132D*), as well as Inositol Polyphosphate-5-Phosphatase A (*INPP5A*), and Transcriptional Adaptor 1 (*TADA1*) for the paired analyses (cross-sectional vs. longitudinal cognitive functioning). For cognition at follow-up, *ZBTB46* and the Src Homology 2 Domain Containing F gene (*SHF*) were common between paired and unpaired analyses, while *SORBS1* was a common gene for change of cognition. An overview of the overlapping probes with corresponding *p*-values and annotations is presented in Table 2, while additionally overlapping genes between analyses are presented in Supplementary Table 3.

**Table 2:** Overlap of associated probes (*p* < 10^−4^) identified from the paired and unpaired EWAS analyses.

		**Probe**	***P*-value (1)**	***P*-value (2)**	**Chr**	**Position (Bp)**	**Gene**	**Distance to Gene (Bp)**	**Feature CGI^c^**
Overlapping findings between EWAS analyses	Overlap paired: cognition (1) vs. cognition change (2)	cg12294361	1.26E-05	6.64E-05	2	67877000	*ETAA1*	25,2558	IGR^a^-open sea
		cg15892650	1.74E-05	4.49E-05	5	150524635	*ANXA6*	NA	Body-open sea
		cg17261234	4.38E-05	3.75E-06	14	101925619	*DIO3OS*	−9,2941	IGR^a^-island
		cg26614129	6.82E-05	3.22E-05	12	130311289	*TMEM132D*	NA	Body-open sea
	Overlap cognition: paired (1) vs. unpaired (2)	cg05867245	5.84E-07	1.20E-05	20	62402415	*ZBTB46*	NA	Body-island
	Overlap cognition difference: paired (1) vs. unpaired (2)	cg25421530	4.79E-05	5.28E-06	10	97200987	*SORBS1*	NA	5′UTR^b^-open sea

a *IGR, Intergenic region*;

b *UTR, Untranslated Region*;

c *CGI, CpG Island*.

Pathway analyses for KEGG and Pathway Commons were performed on all results with *p* < 10^−4^ obtained from both paired and unpaired analyses on cognition at follow-up and change of cognition (Table 3 and Supplementary Table 4). Pathway analysis for KEGG, based on top results from paired analysis of cognition at follow-up, identified “Neuroactive ligand-receptor interaction” (*p* = 0.0098 BH-adjusted *p* = 0.059) and “Gap junction” (*p* = 0.030, BH-adjusted *p* = 0.091) as the most enriched pathways. The most enriched pathways in Pathway Commons analysis, based on top results from paired cognition at follow-up, were “GABA receptor activation” (*p* = 0.0056, BH-adjusted *p* = 0.29) and “Neurotransmitter Receptor Binding And Downstream Transmission In The Postsynaptic Cell” (*p* = 0.032, BH adjusted *p* = 0.54). Enrichment signal in both pathways was driven by the same two genes: GABA(A) Receptor Subunit Gamma-3 (*GABRG3*) and Guanine Nucleotide Binding Protein (G Protein), Alpha Inhibiting Activity Polypeptide 3 (*GNAI3*). Overview of all results obtained from KEGG pathway analyses (pathways with BH-corrected *p* < 0.1) are presented in Table 3 and from Pathway Commons analyses (pathways with unadjusted *p* < 0.05) in Supplementary Table 4.

**Table 3:** Enriched pathways in KEGG (BH-corrected *p* < 0.1) based on associated findings from EWAS analyses (*p* < 10^−4^).

		**Pathway name**	**#Genes**	**Gene symbol**	**Statistics**
Paired	Cognition	Neuroactive ligand-receptor interaction	4	*TAAR8 DRD2 GRM8 GABRG3*	*C* = 268; *O* = 4; *E* = 0.84; *R* = 4.78; raw*P* = 0.0098; adj*P* = 0.0588
		Gap junction	2	*DRD2 GNAI3*	*C* = 87; *O* = 2; *E* = 0.27; *R* = 7.37; raw*P* = 0.0302; adj*P* = 0.0906
		Axon guidance	2	*EFNA5 GNAI3*	*C* = 128; *O* = 2; *E* = 0.40; *R* = 5.01; raw*P* = 0.0606; adj*P* = 0.0909
		Parkinson's disease	2	*NDUFA4 DNCA*	*C* = 114; *O* = 2; *E* = 0.36; *R* = 5.62; raw*P* = 0.0494; adj*P* = 0.0909
Unpaired	Cognition	Basal transcription factors	2	*GTF2H1 TAF12*	*C* = 35; *O* = 2; *E* = 0.05; *R* = 36.63; raw*P* = 0.0014; adj*P* = 0.0028
	Change of cognition	Insulin signaling pathway	2	*RPTOR SORBS1*	*C* = 138; *O* = 2; *E* = 0.14; *R* = 13.93; raw*P* = 0.0089; adj*P* = 0.0089

*C, the number of reference genes in the category; O, the number of genes in the gene set and also in the category; E, the expected number in the category; R, ratio of enrichment; rawP, p-value from hypergeometric test; adjP, p-value adjusted by the multiple test adjustment*.

## Discussion

In this study we investigated if DNA methylation signatures in blood associate with cognitive functioning in middle-aged individuals in a cross-sectional and longitudinal manner. Use of a within-twin-pair design in this study of monozygotic twins allowed us to investigate epigenetic age-related changes in cognitive functioning regardless of the genetic background of the investigated individuals.

Investigation of cross-sectional cognitive functioning in 486 middle-aged twins identified DNA methylation of a CpG site positioned in an intronic CpG island of *ZBTB46* as the most associated finding, which was supported in both the paired 50% most discordant and unpaired analyses, however with higher *p*-values. Higher *p*-value in the 50% most discordant twins analysis in comparison to the full MADT cohort is probably due to decreased number of individuals and thus decreased power of this model. *ZBTB46* is a gene most highly expressed in cerebellum and cerebellar hemisphere, according to the GTEx (Lonsdale et al., 2013). Expression of *Zbtb46* has been also shown to distinguish classical dendritic cells and their committed progenitors from other immune lineages (Satpathy et al., 2012). A genetic polymorphism in *ZBTB46* (rs6062314) was associated with multiple sclerosis (Lill et al., 2013), with the identified SNP located 7298 bp from the *ZBTB46* CpG site associated in our study. Search for the possible methylation Quantitative Trait Loci interactions between rs6062314 and methylation levels in fetal brain samples (Hannon et al., 2016) yielded no results.

Additionally, the unpaired analysis of cross-sectional cognitive functioning identified a probe positioned in a region 1,500 bp upstream from transcription start site of *TAF12* as the most associated finding. One of the best-described functions of *TAF12* is its interaction and recruitment of the growth arrest and DNA damage-inducible alpha protein (Gadd45a) (Schmitz et al., 2009), responsible for active DNA demethylation and known for its important role in neurodevelopment, neuronal injury, and ischemia, where *Gadd45a* mRNA levels are broadly increased throughout the ischemic cortex (Sultan and Sweatt, 2013) in mice. None of our top findings of *p* < 10^−5^ overlapped with the loci associated with non-pathological cognitive ageing in recent genome-wide association studies (Davies et al., 2014, 2015). Pathway analyses of the most associated findings associated with cross-sectional cognition measurement identified “Neuroactive ligand-receptor interaction” as the most enriched pathway suggesting methylation of neurobiology-related genes to be involved in cognitive functioning. A recent animal study, based on methylated DNA immunoprecipitation sequencing method, reported differential methylation in genes enriched in “main axon,” “dendritic spine head,” and “postsynaptic density” Gene Ontology pathways to be associated with associative memory maintenance (Halder et al., 2016). Interestingly, another animal study, based on whole-genome bisulfite sequencing, reported age-related impairment of cognitive flexibility to be associated with differential methylation of genes enriched in “postsynaptic density” Gene Ontology pathways (Ianov et al., 2017). These studies further support that epigenetic changes in genes in neurobiology-related pathways are associated with both cross-sectional, as well as longitudinal cognitive functioning.

In the intra-pair analyses the longitudinal change in cognitive functioning (mainly decline) was most highly associated with a probe positioned in *AGBL4*, also known as *CCP6*. The gene is known for its brain-specific expression, with highest expression levels observed in frontal cortex (Lonsdale et al., 2013) and its function in catalyzing deglutamylation of tubulin. The accumulation of posttranslationally added glutamate side chains at microtubules is directly linked to neurodegeneration, thus highlighting the important role of *AGBL4* for neuronal survival (Rogowski et al., 2010). It is possible that differential methylation of *AGBL4* impacts its expression and further protein levels, what dysregulates the tubulin deglutamylation and leads to neurodegeneration and decrease of cognitive function, however this hypothesis needs to be further tested in translational models of cognitive aging.

Interestingly, the most associated finding of cognitive change in the unpaired analyses was a *SORBS1* probe, also associated with the trait in the paired analysis but with a higher *p*-value. Genetic polymorphism in *SORBS1* has been suggested to be associated with brain infarction related to ischemic stroke. However since the finding was reported in Japanese individuals it should be replicated in other populations to be directly related to our study (Hagiwara et al., 2008). Intriguingly, *SORBS1* expression has been found to be upregulated in the hippocampus of individuals suffering from Alzheimer's disease in comparison to unaffected controls (Blalock et al., 2004), supporting its potential function in cognitive impairment.

A possible limitation of this study is the use of the more easily accessible blood samples to study DNA methylation signatures of a brain-related trait. However, recent studies that investigated the possibility of using blood as a proxy for brain-related phenotypes reported DNA methylation status in brain to be mirrored at many CpG sites in blood, thus supporting the use of more commonly available blood samples for studying mental health (Aberg et al., 2013). It should also be noted that the blood samples used in this study were collected from twins only at the follow-up visit, not on both occasions. Accordingly, we were only able to correlate longitudinal change of cognition with DNA methylation status at follow-up, while we cannot estimate a possible relation between cognition change and ageing-related changes in methylation. This study provides only information on the associations between cognitive functioning and DNA methylation levels in blood. Further research is warranted to answer the question if the associated differences in DNA methylation levels are causal of cognitive functioning or are a consequence of any disease's pathological process (Aberg et al., 2013; Mill and Heijmans, 2013).

In addition, perhaps of particular relevance for the longitudinal measure of cognitive aging identification of an appropriate population for such a study is a challenge, since part of investigated individuals may be too healthy to show any age-related cognitive decline or too ill to be able to perform the test (Minder et al., 2002) resulting in decreased variance in the sample of the studied trait. In our sample we observed a decrease in mean score on the cognition assessment test in comparison to the first visit of investigated twins, however, for some individuals improvement in their cognitive score was recorded during the follow-up assessment. The improvement in the cognition score at follow-up can be linked with possible changes in lifestyle of an individual, practice effect or illness at the first visit. Reduced risk of dementia and cognitive decline incident has been linked with higher levels of education, occupational complexity, cognitive leisure activities, while active cognitive lifestyle was even associated with cognitive recovery (Valenzuela and Sachdev, 2006; Marioni et al., 2012). Additionally, nutrition, level of physical and social activity are well-established lifestyle factors linked to preventing cognitive decline and proposed to be used in interventions to restore and maintain the cognitive level (Williams and Kemper, 2010).

In this study we did not have access to information on medication, which could have an influence on blood DNA methylation levels and cognitive functioning. A recent study showed the use of an approved anti-asthma medication to reduce neuroinflammation, elevate hippocampal neurogenesis and improve learning and memory in old rats, making it highly relevant to include drug information in future studies of DNA methylation and cognitive functioning (Marschallinger et al., 2015). Moreover, level of educational attainment is associated with cross-sectional cognitive functioning, but not with the rate of cognitive decline in aging individuals (Wilson et al., 2009; Zahodne et al., 2011). Data on educational attainment was not available for this study. Future studies adjusting for this variable will delineate to what extend our EWAS findings for cross-sectional measure of cognitive functioning are influenced by the level of education.

In conclusion, this study investigated possible blood DNA methylation signatures related to cross-sectional and longitudinal cognitive functioning in middle-aged individuals and identified associations with this epigenetic modification of neuronal survival and ischemia-related genes. In the future, in order to get a deeper insight into the mechanisms related to cognitive aging, replication and follow-up of these findings in an independent population is warranted, preferably using both blood and more biologically cognition-related tissue, like frontal cortex or hippocampus.

## Author contributions

Conception and design: AS, QT, LC, and MM; Analysis: AS, QT, and LC; Interpretation: AS, QT, MM, OM, AB, KC, MN, and LC; Drafting the manuscript: AS, QT, MN, and LC; Revising the article and final approval of the version to be published: AS, QT, MM, OM, AB, KC, MN, and LC.

### Conflict of interest statement

The authors declare that the research was conducted in the absence of any commercial or financial relationships that could be construed as a potential conflict of interest.
