# Supplementary material for: Epigenome-Wide Association Study of Cognitive Functioning in Middle-Aged Monozygotic Twins
Source: Front Aging Neurosci. 2017 Dec 12;9:413. doi: 10.3389/fnagi.2017.00413 (PMC5733014; doi:10.3389/fnagi.2017.00413)
Supplement: Supplementary file 1 [file Image1.PDF]

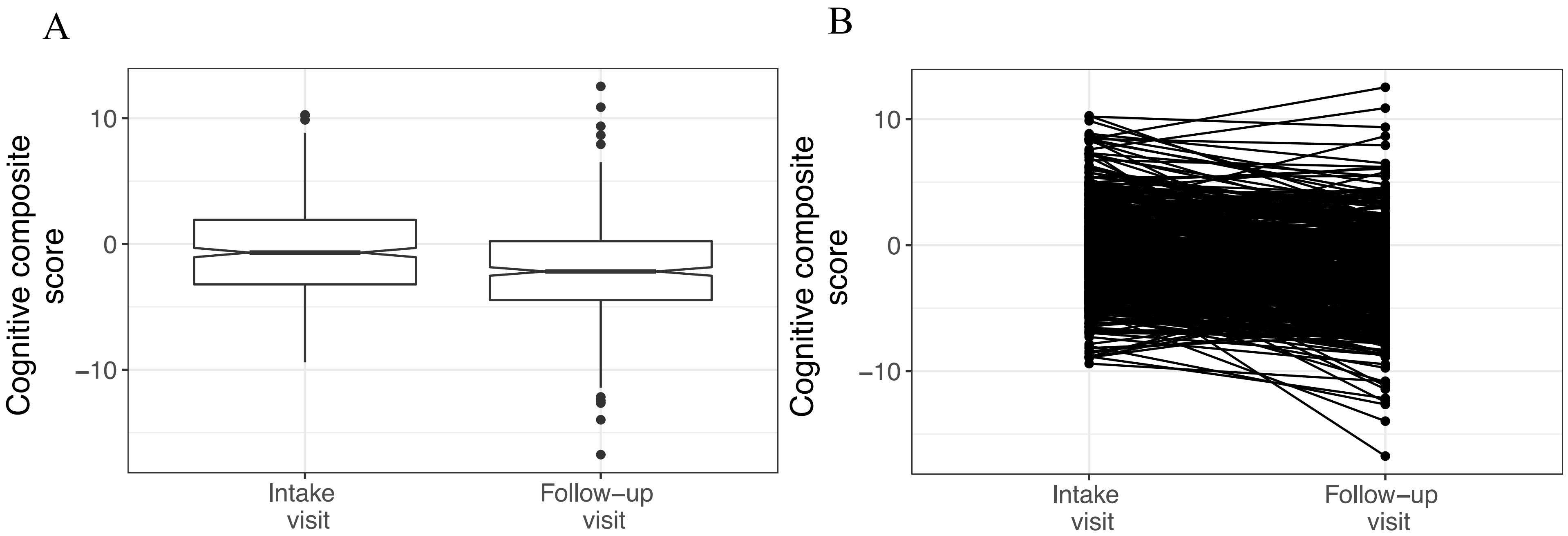

Supplementary Figure 1. Overview of cognitive composite score at intake and follow-up visit visualized for the whole monozygotic twins cohort (Panel A), as well as for each individual (Panel B)
