# Supplementary figures and images for: Epigenome-Wide Association Study of Cognitive Functioning in Middle-Aged Monozygotic Twins
Source: Front Aging Neurosci. 2017 Dec 12;9:413. doi: 10.3389/fnagi.2017.00413 (PMC5733014; doi:10.3389/fnagi.2017.00413)

Overlap of probes, p-value < 0.0001

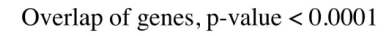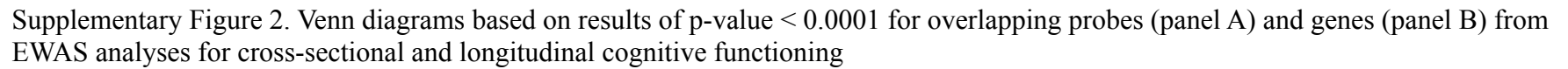

Supplement: Supplementary file 2 [file Image2.PDF]
