# Supplementary material for: Epigenome-Wide Association Study of Cognitive Functioning in Middle-Aged Monozygotic Twins
Source: Front Aging Neurosci. 2017 Dec 12;9:413. doi: 10.3389/fnagi.2017.00413 (PMC5733014; doi:10.3389/fnagi.2017.00413)
Supplement: Supplementary file 3 [file Table1.DOCX]

Supplementary Table 1. Demographics of the cohort of monozygotic twin pairs included in this study

| **Variable** | **Females** | **Males** | **All** |
| --- | --- | --- | --- |
| **Number of individuals** | 222 | 264 | 486 |
| **Mean age at intake (years)** | 54.8 ±6.0 | 55.5 ±6.1 | 55.2 ±6.0 |
| **Mean age at follow-up (years)** | 65.6 ±6.0 | 66.2 ±6.1 | 65.9 ±6.1 |
| **Mean cognitive composite score at intake** | 0.4 ±3.6 | -1.3 ±3.6 | -0.5 ±3.7 |
| **Mean cognitive composite score at follow-up** | -1.3 ±3.6 | -2.9 ±3.7 | -2.2 ±3.8 |
