# Supplementary material for: Epigenome-Wide Association Study of Cognitive Functioning in Middle-Aged Monozygotic Twins
Source: Front Aging Neurosci. 2017 Dec 12;9:413. doi: 10.3389/fnagi.2017.00413 (PMC5733014; doi:10.3389/fnagi.2017.00413)
Supplement: Supplementary file 4 [file Table2.DOCX]

Supplementary Table 2. Probes identified in both: all twins and 50% most discordant twins pairs in the paired EWAS on cross-sectional cognition (p-value < 10^-4^)

|  | | | | | | | | | |
| --- | --- | --- | --- | --- | --- | --- | --- | --- | --- |
| **Probe** | **P-value (all)** | **P-value**  **(most discordant)** | **Chr** | **Position (Bp)** | **Gene** | **Distance to Gene (Bp)** | **Gene feature** | **CGI** | **Feature CGI**^c^ |
| **cg05867245** | 5.84E-07 | 2.45E-05 | 20 | 62402415 | *ZBTB46* | NA | Body | island | Body - island |
| **cg07157058** | 5.35E-05 | 6.96E-06 | 6 | 7390083 | *CAGE1* | NA | TSS200 | island | TSS^b^200 - island |
| **cg13686863** | 1.63E-05 | 1.98E-05 | 6 | 132872645 | *TAAR8* | NA | TSS1500 | open sea | TSS1500 - open sea |
| **cg17261234** | 4.38E-05 | 7.22E-06 | 14 | 101925619 | *DIO3OS* | -92941 | IGR | island | IGR^a^ - island |

IGR^a^: Intergenic Region, TSS^b^: Transcription Start Site, CGI^c^: CpG Island
