# Supplementary material for: Epigenome-Wide Association Study of Cognitive Functioning in Middle-Aged Monozygotic Twins
Source: Front Aging Neurosci. 2017 Dec 12;9:413. doi: 10.3389/fnagi.2017.00413 (PMC5733014; doi:10.3389/fnagi.2017.00413)
Supplement: Supplementary file 5 [file Table3.DOCX]

Supplementary Table 3. Loci where two separate probes from different EWAS analyses map the same gene.

|  |  | **Gene name** | **Probe (1)** | **P-value (1)** | **Probe (2)** | **P-value (2)** |
| --- | --- | --- | --- | --- | --- | --- |
| **Additionally overlapping genes** | **Overlap paired: cognition (1) vs cognition change (2)** | *INPP5A* | cg25745246 | 7.31E-05 | cg02483043 | 3.86E-06 |
|  |  | *TADA1* | cg05016425 | 8.67E-05 | cg15453345 | 7.61E-05 |
|  | **Overlap cognition: paired(1) vs unpaired (2)** | *SHF* | cg01908020 | 2.35E-05 | cg13580343 | 2.88E-05 |

(1) and (2) numbering correspond to the EWAS analysis presented in a given row.
