# Supplementary material for: Epigenome-Wide Association Study of Cognitive Functioning in Middle-Aged Monozygotic Twins
Source: Front Aging Neurosci. 2017 Dec 12;9:413. doi: 10.3389/fnagi.2017.00413 (PMC5733014; doi:10.3389/fnagi.2017.00413)
Supplement: Supplementary file 6 [file Table4.DOCX]

Supplementary Table 4. Enriched pathways in Pathway Commons (unadjusted p-value < 0.05) based on findings from EWAS analyses (p-value < 10^-4^)

|  |  | Pathway name | #Gene | Gene name | Statistics |
| --- | --- | --- | --- | --- | --- |
| Paired | Cognition | GABA receptor activation | 2 | *GNAI3 GABRG3* | C=36;O=2;E=0.11;R=17.80;rawP=0.0056;adjP=0.2856 |
|  |  | Neurotransmitter Receptor Binding And Downstream Transmission In The Postsynaptic Cell | 2 | *GNAI3 GABRG3* | C=89;O=2;E=0.28;R=7.20;rawP=0.0315;adjP=0.5355 |
|  |  | FOXA transcription factor networks | 2 | *PISD ALAS1* | C=81;O=2;E=0.25;R=7.91;rawP=0.0265;adjP=0.5355 |
|  | Change of cognition | Signaling events mediated by the Hedgehog family | 2 | *SAFT ADRBK1* | C=65;O=2;E=0.21;R=9.54;rawP=0.0187;adjP=0.6250 |
|  |  | Potassium Channels | 2 | *KCNK10 KCNMA1* | C=76;O=2;E=0.25;R=8.16;rawP=0.0250;adjP=0.6250 |
| Unpaired | Cognition | RNA Polymerase II Transcription Initiation | 2 | *GTF2H1 TAF12* | C=38;O=2;E=0.06;R=33.74;rawP=0.0016;adjP=0.0149 |
|  |  | RNA Polymerase II Promoter Escape | 2 | *GTF2H1 TAF12* | C=38;O=2;E=0.06;R=33.74;rawP=0.0016;adjP=0.0149 |
|  |  | RNA Polymerase II Transcription Initiation And Promoter Clearance | 2 | *GTF2H1 TAF12* | C=38;O=2;E=0.06;R=33.74;rawP=0.0016;adjP=0.0149 |
|  |  | HIV-1 Transcription Initiation | 2 | *GTF2H1 TAF12* | C=38;O=2;E=0.06;R=33.74;rawP=0.0016;adjP=0.0149 |
|  |  | RNA Polymerase II Transcription Pre-Initiation And Promoter Opening | 2 | *GTF2H1 TAF12* | C=38;O=2;E=0.06;R=33.74;rawP=0.0016;adjP=0.0149 |
|  |  | RNA Polymerase II HIV-1 Promoter Escape | 2 | *GTF2H1 TAF12* | C=38;O=2;E=0.06;R=33.74;rawP=0.0016;adjP=0.0149 |
|  |  | RNA Polymerase II Pre-transcription Events | 2 | *GTF2H1 TAF12* | C=57;O=2;E=0.09;R=22.49;rawP=0.0036;adjP=0.0266 |
|  |  | Transcription of the HIV genome | 2 | *GTF2H1 TAF12* | C=59;O=2;E=0.09;R=21.73;rawP=0.0038;adjP=0.0266 |
|  |  | Metabolism of lipids and lipoproteins | 3 | *ACADM PLPP3 ABCC1* | C=250;O=3;E=0.39;R=7.69;rawP=0.0068;adjP=0.0423 |
|  |  | Late Phase of HIV Life Cycle | 2 | *GTF2H1 TAF12* | C=91;O=2;E=0.14;R=14.09;rawP=0.0088;adjP=0.0448 |
|  | Change of cognition | ErbB1 downstream signaling | 4 | *RPTOR SORBS1 FGF23 CD82* | C=1254;O=4;E=1.30;R=3.07;rawP=0.0377;adjP=0.0462 |
|  |  | Arf6 signaling events | 4 | *RPTOR SORBS1 FGF23 CD82* | C=1254;O=4;E=1.30;R=3.07;rawP=0.0377;adjP=0.0462 |
|  |  | Syndecan-1-mediated signaling events | 4 | *RPTOR SORBS1 FGF23 CD82* | C=1266;O=4;E=1.32;R=3.04;rawP=0.0388;adjP=0.0462 |
|  |  | Sphingosine 1-phosphate (S1P) pathway | 4 | *RPTOR SORBS1 FGF23 CD82* | C=1277;O=4;E=1.33;R=3.01;rawP=0.0399;adjP=0.0462 |
|  |  | Signaling events mediated by VEGFR1 and VEGFR2 | 4 | *RPTOR SORBS1 FGF23 CD82* | C=1262;O=4;E=1.31;R=3.05;rawP=0.0385;adjP=0.0462 |
|  |  | Arf6 trafficking events | 4 | *RPTOR SORBS1 FGF23 CD82* | C=1254;O=4;E=1.30;R=3.07;rawP=0.0377;adjP=0.0462 |
|  |  | LKB1 signaling events | 4 | *RPTOR SORBS1 FGF23 CD82* | C=1274;O=4;E=1.33;R=3.02;rawP=0.0396;adjP=0.0462 |
|  |  | Class I PI3K signaling events | 4 | *RPTOR SORBS1 FGF23 CD82* | C=1254;O=4;E=1.30;R=3.07;rawP=0.0377;adjP=0.0462 |
|  |  | ErbB receptor signaling network | 4 | *RPTOR SORBS1 FGF23 CD82* | C=1277;O=4;E=1.33;R=3.01;rawP=0.0399;adjP=0.0462 |
|  |  | Class I PI3K signaling events mediated by Akt | 4 | *RPTOR SORBS1 FGF23 CD82* | C=1254;O=4;E=1.30;R=3.07;rawP=0.0377;adjP=0.0462 |

C: the number of reference genes in the category; O: the number of genes in the gene set and also in the category; E: the expected number in the category; R: ratio of enrichment; rawP: p value from hypergeometric test; adjP: p-value adjusted by the multiple test adjustment
